# Effects of Cisplatin on the Radiation Response and DNA Damage Markers in Peripheral Blood Lymphocytes Ex Vivo

**DOI:** 10.3390/cells14100682

**Published:** 2025-05-08

**Authors:** Sebastian Zahnreich, Aisha Bhatti, Barea Ahmad, Sophia Drabke, Justus Kaufmann, Heinz Schmidberger

**Affiliations:** Department of Radiation Oncology and Radiation Therapy, University Medical Center of the Johannes Gutenberg University Mainz, 55131 Mainz, Germany

**Keywords:** ionizing radiation, cisplatin, radiotherapy, peripheral blood lymphocytes, DNA damage, micronuclei, apoptosis, lymphopenia, biodosimetry

## Abstract

Platinum-based radiochemotherapy is associated with hematologic side effects, impacting patient outcomes. However, the clinical mechanisms of cisplatin and its interaction with ionizing radiation (IR), including in biodosimetry for radiotherapy, have not yet been fully clarified. For this purpose, healthy donors’ peripheral blood lymphocytes (PBLs) were pretreated with cisplatin in a pulse (1–4 h) or continuous (24 h) regimen followed by X-rays. DNA damage was assessed as DNA double-strand breaks using repair foci of γH2AX and 53BP1 after 0.5 h and 24 h in G1 PBLs and a proliferation-based cytokinesis-block micronucleus assay. Additionally, cell death and proliferation activity were measured. Unlike a 1 h pulse, a 24 h cisplatin pretreatment caused a concentration-dependent increase in cisplatin-induced foci while decreasing IR-induced foci, especially 24 h after irradiation. This was accompanied by increased apoptosis, with cisplatin and IR having additive effects. Both genotoxins alone caused a dose-dependent increase in micronuclei, while cisplatin significantly reduced binuclear cells, especially after the 24 h treatment, leading to lower micronuclei frequencies post-irradiation. Our results show that prolonged cisplatin exposure, even at low concentrations, impacts the vitality and division activity of PBLs, with significantly stronger effects post-irradiation. This has major implications and must be considered for the detection of DNA damage-associated biomarkers in PBLs used in clinical prediction or biodosimetry during radiotherapy.

## 1. Introduction

Chemotherapy (CT) with cisplatin (cis-diamminedichloroplatinum II (CDDP)) has been used to treat cancer patients since the early 1970s [1] and emerged as a standard of care for a variety of tumor entities, including leukemia, lymphoma, breast, testicular, ovarian, head and neck, and cervical cancer and sarcomas [2]. Subsequent combinations with radiotherapy (RT) as a neoadjuvant, primary, or adjuvant concurrent chemoradiotherapy (CRT) has drastically improved clinical outcomes through spatial cooperation, cytotoxic enhancement, biological cooperation, temporary modulation, and normal tissue protection [3,4]. Concurrent CRT with CDDP achieves additive or synergistic anti-neoplastic effects for many locally advanced (LA) tumors with good locoregional control and acceptable toxicity profiles [2].

CDDP and ionizing radiation (IR) both have DNA-damaging effects. IR induces a large variety of genomic lesions, such as base damage and single-strand breaks (SSBs) or DNA double-strand breaks (DSBs). The main target of CDDP is also considered to be nuclear DNA through the induction of inter- and intrastrand cross-links, although just about 1% of intracellular CDDP reacts with genomic DNA [5]. After cellular uptake, CDDP is aquatized, and its platinum atom covalently binds to the N^7^ position of purine bases. This forms ~65% GpG and 25% ApG 1,2-intrastrand cross-links, ~5–10% GpNpG 1,3-intrastrand cross-links, and a smaller fraction of interstrand cross-links [6]. CDDP-induced DNA adducts can excerpt a radiosensitizing effect by turning IR-induced sublethal DNA damage into irreparable lesions leading to cytogenetic damage, cell cycle arrest, and apoptosis [7,8,9]. This response has also been attributed to the CDDP-mediated inhibition of non-homologous end joining (NHEJ) as the predominant mechanism of DSB repair [10,11]. Moreover, CDDP also has effects on mitochondria, reactive oxygen species, lysosomes, and calcium signaling, which contributes to the synergies with IR for cytotoxic effects [12]. However, the precise molecular mechanisms of CDDP and their interactions with each other and IR remain largely unclear [13]. While a combined effect in the tumor is desired, toxicities in normal tissue should be avoided as far as possible, which are generally higher with concomitant CRT than with the respective monotherapies [14,15]. This applies not only to normal solid tissue but also to the hematologic system of cancer patients, which is inevitably exposed to the systemic effects of CDDP administered intravenously and IR during external beam RT. Lymphocytes in particular, as the main players in the inflammatory anti-tumor response, are highly susceptible to adverse effects of genotoxic cancer therapies [16,17]. Grade III/IV lymphopenia is a common side effect of CRT and a poor prognostic factor for many solid tumor types [18]. Also, RT exposure of tumor-draining lymph nodes impairs T-cell priming and maturation of cytotoxic T cells [19,20]. Although these effects of genotoxic therapies on the hematologic system have been long known, they are gaining increasing attention in light of multimodal cancer therapies with immune checkpoint inhibitors (ICIs) [21,22,23]. At present, the failure of multimodal therapies with ICIs is primarily attributed to the adverse effects of genotoxic therapies on the hematologic system, which is crucial to the patient’s inherent anti-tumor immune response [19,24]. A key example is LA head and neck tumors, where the still generally poor survival rates in concurrent CRT could not be improved by immuno-oncological strategies with ICIs [25,26,27].

The radiation burden on the hematological system of cancer patients can be monitored in vivo using biodosimetric markers that are primarily based on the detection of IR-induced DNA damage in peripheral blood lymphocytes (PBLs) [28,29,30]. The most common assays are immunodetection of nuclear foci of DNA repair proteins γH2AX or 53BP1 as DSB surrogate markers, chromosomal aberrations, or micronuclei [31]. The effects of a concomitant CT on these radiobiological endpoints, in particular, the role of CDDP as a potential confounder and its impact on the sensitivity of PBL to IR, have not been thoroughly investigated to date. Scarcely available studies indicate an impairment of the IR-induced DSB response, stronger cell cycle arrest, and increased apoptosis in PBLs of cancer patients who received concomitant CRT with CDDP [32,33,34].

To increase the understanding of the effects of a combination of genotoxic therapies on the hematological system, we examined the ex vivo effects of CDDP pretreatments and subsequent IR exposure of isolated PBLs from buffy coats of healthy donors on the induction and repair of DSBs, micronuclei formation, cell cycle, and apoptosis. The data generated provide detailed information on the confounding effect of cytostatic drugs on these endpoints used as predictive biomarkers and for biodosimetry during RT and the adverse effects of corresponding therapeutic combinations on the hematological system of tumor patients.

## 2. Materials and Methods

### 2.1. Isolation of Peripheral Blood Mononuclear Cells

Buffy coats of leukocyte-rich plasma of healthy human donors were kindly provided by the Institute for Transfusion Medicine—Transfusion Centre of the University Medical Center Mainz. Ethical approval was obtained from the Medical Association of Rhineland-Palatinate [no. 2023-17191 and 2024-17401_2], and all research was performed following relevant guidelines and regulations. All donors provided informed consent, and this research was performed following the Declaration of Helsinki. A total of 1–2 mL of the buffy coats was diluted with 3–4 mL of the X-VIVO^TM^ 15 medium (Lonza Group Ltd., Basel, Switzerland), and peripheral blood mononuclear cells (PBMCs) were separated by Histopaque (Histopaque 1077, Sigma-Aldrich, Taufkirchen, Germany) density gradient centrifugation, as described previously [28,35,36]. We obtained an average of 7–8 × 10^6^ PBMCs per mL of buffy coat. Isolated PBMCs were washed once in 2 mM of ethylenediaminetetraacetic acid (EDTA, Merck, Germany)/1× a phosphate-buffered saline (PBS, Merck, Germany); resuspended in the X-VIVO^TM^ 15 medium; counted; and used for assays. For logistical reasons, it was not always possible to use the same buffy coat across all assays.

### 2.2. Irradiation

Cells were exposed to X-rays (140 kV) at room temperature using the D3150 X-ray Therapy System (Gulmay Medical Ltd., Byfleet, UK) at a dose rate of 3.6 Gy/min or were sham-irradiated (0 Gy), i.e., kept for the same time in the radiation device control room.

### 2.3. γH2AX and 53BP1 Focus Quantification

A total of 1–2 × 10^6^ PBMCs were diluted in 5 mL of the X-VIVO^TM^ 15 medium. CDDP (Accord Healthcare GmbH, München, Germany) was added at concentrations of 0, 1, 5, 10, and 20 µg/mL, and cells were incubated in a humidified incubator at 5% CO_2_ and 37 °C. After 1 h or 24 h, the medium was replaced with a CDDP-free X-VIVO^TM^ 15 medium, and the cells were irradiated with 0.5 Gy or 2 Gy X-rays. The samples irradiated with 0.5 Gy or 2 Gy were processed after 0.5 h or 24 h, respectively. A sham-irradiated sample (0 Gy) was included in each case. Samples with irradiation only were treated accordingly without CDDP. Fixation of PBLs, γH2AX and 53BP1 immunostaining, fluorescence microscopy, image capturing, and foci scoring were performed as described previously [28,35,36,37]. Only a pair of colocalizing γH2AX and 53BP1 foci was considered a true DSB focus to avoid false positives. Three independent repeat experiments were carried out with individual samples for all experimental conditions. For each data point, at least 100 sham-irradiated cells, 50 cells at 0.5 h post-irradiation, and 100 cells at 24 h post-irradiation were analyzed.

### 2.4. Cytokinesis Block Micronucleus (CBMN) Assay

A total of 3 × 10^6^ cells were diluted in 5 mL of the X-VIVO^TM^ 15 medium containing a 20% heat-inactivated fetal calf serum (FCS, Biochrom, Berlin, Germany) and 1% phytohemagglutinin (PHA, Thermo Fisher Scientific, Karlsruhe, Germany) to simulate proliferation. CDDP was added at concentrations of 0, 1, 2.5, 5, 7.5, 10, and 20 µg/mL, and cells were incubated in a humidified incubator at 5% CO_2_ and 37 °C. After 4 h or 24 h, the medium was replaced with a CDDP-free X-VIVO^TM^ 15 medium containing a 20% heat-inactivated FCS and 1% PHA. Cells were irradiated with 2 Gy X-rays and incubated in a humidified incubator at 5% CO_2_ and 37 °C for up to 72 h after the start of cultivation. Samples with irradiation only were treated accordingly without CDDP. A total of 6 µg/mL cytochalasin B (Biomol, Hamburg, Germany) was added 24 h after the start of incubation to abrogate cytokinesis and accumulate binucleated (BN) cells during the remaining 48 h incubation period. Cells were spun down; treated for 10 min with 0.075 M of a potassium chloride solution (Merck, Darmstadt, Germany) for hypotonic shock; and fixed twice with Carnoy’s solution. Cells were dropped on slides and mounted with a HOECHST33258 antifade mountant. Image acquisition by fluorescence microscopy and automated scoring of micronuclei (MN) in BN cells were performed using the Metafer platform and the MNScore X software (Metasystems, Altlussheim, Germany). The binucleation index was calculated as the ratio of the number of BN cells/total number of cells (mononucleated + BN cells). On average, 38,747 ± 10,260 total cells and 3854 ± 1426 BN cells were analyzed per sample to evaluate MN and the binucleation index. To determine the number of radiation-induced MN, the corresponding basal values were subtracted. Three independent repeat experiments were carried out with single samples for all experimental conditions.

### 2.5. Apoptosis (Annexin V-FITC/PI Staining)

For apoptosis measurement, 1 × 10^6^ PBMCs were diluted in 5 mL of the X-VIVO^TM^ 15 medium. CDDP was added at concentrations of 0, 1, 5, 10, and 20 µg/mL, and cells were incubated in a humidified incubator at 5% CO_2_ and 37 °C. After 24 h, the medium was replaced with a CDDP-free X-VIVO^TM^ 15 medium, and the cells were sham-irradiated (0 Gy) or exposed to X-rays. The cells were incubated in a humidified incubator at 5% CO_2_ and 37 °C for another 24 h. Samples with irradiation only were treated accordingly without CDDP. The fractions of viable, early-apoptotic, late-apoptotic, and necrotic cells were assessed using an Annexin V-FITC/PI detection kit (Miltenyi Biotec, Bergisch Gladbach, Germany), according to the manufacturer’s instructions. At least 10,000 cells per sample were measured in a BD FACSCanto II flow cytometer (BD Biosciences, Billerica, MA, USA). The data were analyzed using the Flowing Software (flowingsoftware.btk.fi). At least three independent experiments with biological duplicates or triplicates were performed for all experimental conditions.

### 2.6. Cell Cycle Measurement

To investigate the effects of CDDP on the cell cycle of PBLs, 1 × 10^6^ cells were diluted in 5 mL of the X-VIVO^TM^ 15 medium containing a 20% heat-inactivated FCS and 1% PHA to stimulate proliferation. At the same time, CDDP treatment was started with concentrations of 0, 1, 5, 10, and 20 µg/mL. After 24 h of incubation in a humidified incubator at 5% CO_2_ and 37 °C, the medium was replaced with a CDDP-free X-VIVO^TM^ 15 medium containing a 20% heat-inactivated FCS and 1% PHA. In a separate experimental setting, an additional 100 ng/mL of nocodazole (Thermo Scientific Chemicals, Karlsruhe, Germany) was added to accumulate mitotic cells. This approach was carried out to monitor cell cycle progression into mitosis, focusing solely on the first cell cycle post-treatment and simulating conditions similar to the CBMN assay. After a further 48 h of incubation, the cells were washed once in 2 mM of EDTA/1× PBS; fixed in 70% Ethanol at −20 °C; treated with RNaseA (Merck, Darmstadt, Germany); and stained with propidium iodide (PI, Merck, Darmstadt, Germany). At least 10,000 cells per sample were analyzed in a BD FACSCanto II flow cytometer (BD Biosciences, Billerica, MA, USA). Quantification of the fractions of cells in G1, S, and G2/M was performed using the Flowing Software (https://flowingsoftware.com/). At least three independent experiments with biological triplicates were performed for all experimental conditions.

### 2.7. Data and Statistical Analyses

Summarized average yields and data are provided as the mean ± standard deviation unless stated otherwise. Data handling, plotting, and statistics were conducted using SigmaPlot Version 14 (Systat Software, USA). The numbers of foci per cell, MN per BN cell, and fractions of dead cells were evaluated and presented as follows: (I) sham-irradiated yield without and with CDDP pretreatment (0 Gy total), (II) observed total yield post-irradiation without and with CDDP pretreatment (X Gy total), (III) expected additive total yield (X Gy total without CDDP + 0 Gy total with CDDP), and (IV) radiation-induced only without and with CDDP pretreatment (X Gy total − 0 Gy total). The relationship between two variables was analyzed using the Pearson test. For a comparison of the means of two or more groups, Student’s *t*-test or the one-way analysis of variance (ANOVA) was used, respectively. To test for the heterogeneity of the slopes of linear regressions, the analysis of covariance (ANCOVA) was applied. All levels of significance were set to α = 0.05.

## 3. Results

### 3.1. DSB Repair Foci

**Figure 1:**
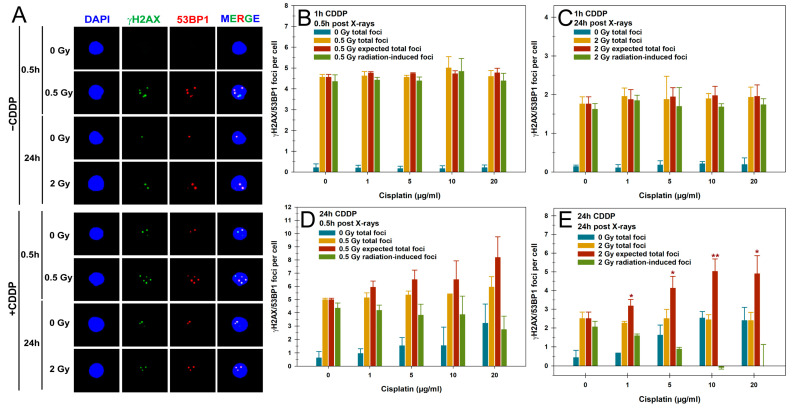
Quantification of colocalized γH2AX and 53BP1 (γH2AX/53BP1) foci in peripheral blood lymphocytes (PBLs) after cisplatin (CDDP), X-rays, or their combination, with a CDDP pretreatment before irradiation. (**A**) Representative fluorescence microscopic images of γH2AX/53BP1 foci in PBLs at 0.5 h and 24 h post-irradiation, without or with 24 h of a 10 µg/mL CDDP pretreatment. Quantification of γH2AX/53BP1 foci in PBLs with (**B**,**C**) a 1 h pulse or (**D**,**E**) 24 h continuous CDDP pretreatment (**B**,**D**) 0.5 h or (**C**,**D**) 24 h after X-ray exposure with 0.5 Gy or 2 Gy, respectively. Focus yields are presented as sham-irradiated without and with CDDP pretreatment (0 Gy total foci), total foci post-irradiation without and with CDDP pretreatment (0.5 or 2 Gy total foci), expected total foci (0.5 or 2 Gy total foci without CDDP + 0 Gy total foci with CDDP), and only radiation-induced foci without and with CDDP pretreatment (0.5 or 2 Gy total foci − 0 Gy total foci). Data are presented as means and standard deviations from three independent experiments with single samples. A statistical comparison was conducted between observed totals post-irradiation (0.5 or 2 Gy total foci) and the expected totals (0 Gy total foci with CDDP + 0.5 or 2 Gy total foci without CDDP) using Student’s *t*-test (* *p* < 0.05; ** *p* < 0.01).

We first examined the impact of CDDP pretreatment on IR-induced DSBs and their repair in unstimulated PBLs in G1 using the γH2AX/53BP1 focus assay. Exemplary fluorescence microscopy images of γH2AX/53BP1 foci in PBLs are shown in Figure 1A for the different treatments. The initial γH2AX/53BP1 radiation-induced foci (RIF) were analyzed 0.5 h post-irradiation with 0.5 Gy, while residual RIF were assessed 24 h after 2 Gy, following either a 1 h or 24 h CDDP pretreatment (Figure 1B–E). Different IR doses ensured countable foci numbers in PBLs at different examination times [28].

Pulse treatment of PBLs with increasing concentrations of CDDP alone for 1 h did not alter the levels of γH2AX/53BP1 foci compared to mock-treated cells, with a total average of 0.19 ± 0.02 focus per PBL (Figure 1B). A total of 0.5 h after 0.5 Gy X-rays, a comparable average of 4.64 ± 0.19 γH2AX/53BP1 total foci per PBL was observed, regardless of CDDP concentration (Figure 1B). This level was only slightly and not significantly lower than the average 4.76 ± 0.02 expected total γH2AX/53BP foci per PBL 0.5 h after 0.5 Gy X-rays (Figure 1B). Similarly, the levels of initial RIF with an average of 4.48 ± 0.20 per cell were also in this range and were comparable between all concentrations of CDDP pulse treatment (Figure 1B). Also, a 24 h incubation of PBLs after a 1 h pulse treatment and 2 Gy X-rays showed no impact on the average basal level of 0.17 ± 0.05 focus per cell following CDDP treatment alone, nor on the average total residual foci and RIF after irradiation of 2.01 ± 0.29 and 1.92 ± 0.11 per cell, respectively (Figure 1C). Thus, no deviation was observed between the total foci and the expected total foci for this treatment regimen. The observed basal foci and RIF values in PBLs closely match our extensive previous γH2AX foci data sets from 14 healthy donors and more than 150 patients with benign or malignant pathologies for similar doses and time points [28,35,36]. Together, short-term pulse treatment with CDDP did not impact initial and residual levels of basal or IR-induced γH2AX/53BP1 foci in PBLs, including at a higher IR dose of 1 Gy (Supplementary Figure S1). The total foci numbers detected remained within the expected range.

Given this finding and our interest in prolonged CDDP exposure effects on PBLs, we switched to a continuous 24 h CDDP pretreatment before irradiation. A total of 24 h of CDDP pretreatment alone caused a concentration-dependent increase in γH2AX/53BP1 foci, peaking at 3.21 ± 1.45 foci per PBL at 20 µg/mL of CDDP (Figure 1D). However, 0.5 h after 0.5 Gy exposure, a similar result of 5.4 ± 0.37 total foci per PBL was observed across all CDDP concentrations, leading to a non-significant underestimation of the expected total foci due to rising CDDP-dependent basal foci levels (Figure 1D). These effects also caused a non-significant decrease in RIF per PBL, depending on the CDDP concentration compared to 0.5 Gy X-rays alone (Figure 1D). Prolonged incubation to assess residual RIF 24 h after 2 Gy exposure also led to a significant increase in mean CDDP-induced foci from 5 µg/mL onward (*p* < 0.05), saturating at 2.46 ± 0.09 foci per PBL at higher concentrations (Figure 1E). As with the early post-irradiation analysis, a comparable average of 2.42 ± 0.10 total foci per PBL was observed across all CDDP concentrations 24 h after 2 Gy exposure, resulting in significant underestimates of the expected total foci numbers from 1 µg/mL of CDDP onward (Figure 1E). This was accompanied by a significant reduction in residual RIF per PBL from 5 µg/mL of CDDP onward (*p* < 0.05), with some values even turning negative at higher CDDP concentrations (Figure 1E).

Notably, a 24 h CDDP treatment and, in particular, the combination with IR led to an increase in cells with pan-nuclear γH2AX signals, a strong indicator of DNA fragmentation during apoptosis (Supplementary Figure S2).

### 3.2. Apoptosis

**Figure 2:**
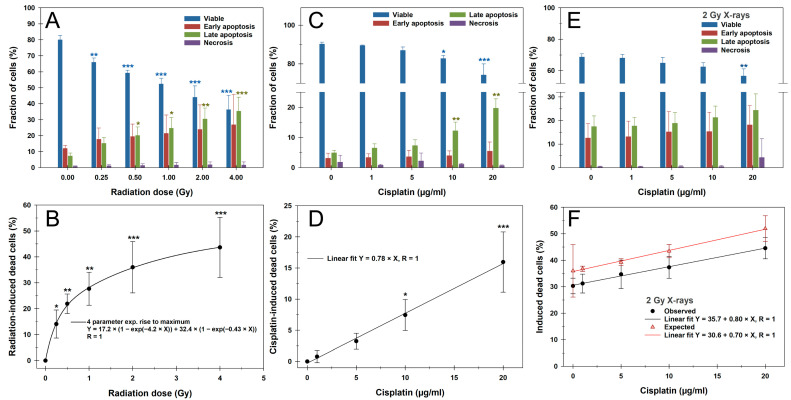
Apoptosis measurement by Annexin V-FITC/PI staining and flow cytometry of peripheral blood lymphocytes after (**A**,**B**) X-rays, (**C**,**D**) cisplatin, or (**E**,**F**) their combination, as a 24 h continuous cisplatin pretreatment before 2 Gy X-rays. (**A**,**C**,**E**) The upper panel shows the fractions of viable, early-apoptotic, late-apoptotic, and necrotic cells. (**B**,**D**,**F**) The lower panel presents radiation- or cisplatin-induced cell death, summing early-apoptotic, late-apoptotic, and necrotic cells, based on radiation dose or cisplatin concentration. The expected proportions of dead cells in F represent the sum of 2 Gy X-rays alone (**A**,**B**) and cisplatin treatment alone (**C**,**D**). The data are presented as mean values and standard deviations of three independent experiments with biological duplicates or triplicates. Statistics were performed by (**A**–**D**) a one-way ANOVA comparison to the mock-treated control (0 Gy; 0 µg/mL CDDP) and (**F**) ANCOVA for the slopes of linear regressions (* *p* < 0.05; ** *p* < 0.01; *** *p* < 0.001).

Based on these observations, we conducted cell death measurements in G1 PBLs 24 h after treatment using the Annexin V/PI assay, as shown in Figure 2. Representative flow cytometric cytograms are provided in Supplementary Figure S3. A total of 24 h after irradiation with X-rays of up to 4 Gy, a significant dose-dependent increase in the proportion of dead cells comprising early and late apoptosis plus necrosis was observed from the lowest dose of 0.25 Gy (Figure 2A,B). Early and late apoptosis occurred in equal proportions, while necrosis was generally negligible for all treatments. Following a steep increment of up to 1 Gy, the fraction of dead cells leveled off and was best fitted with a four-parameter exponential rise to maximum function.

A 24 h treatment with CDDP, followed by 24 h of incubation after CDDP withdrawal, caused a concentration-dependent linear increase in dead cells, with significant proportions from a concentration of 10 µg/mL (Figure 2C,D). Here, late apoptosis dominated in particular. After a 2 Gy irradiation of PBLs pretreated with CDDP for 24 h followed by 24 h of incubation without CDDP, a CDDP-dependent linear increase in dead cells was again observed (Figure 2E,F). A significant difference from irradiation alone was only present at the highest CDDP concentration of 20 µg/mL (*p* < 0.01). The slope of the linear regression line was similar to that of CDDP treatment alone, suggesting an additive effect with IR. Compared to the expected proportion of apoptotic cells based on the additive effects of IR and CDDP alone, the observed yields after the combined treatment were slightly, but not significantly, lower (Figure 2F).

### 3.3. CBMN Assay and Cell Cycle

**Figure 3:**
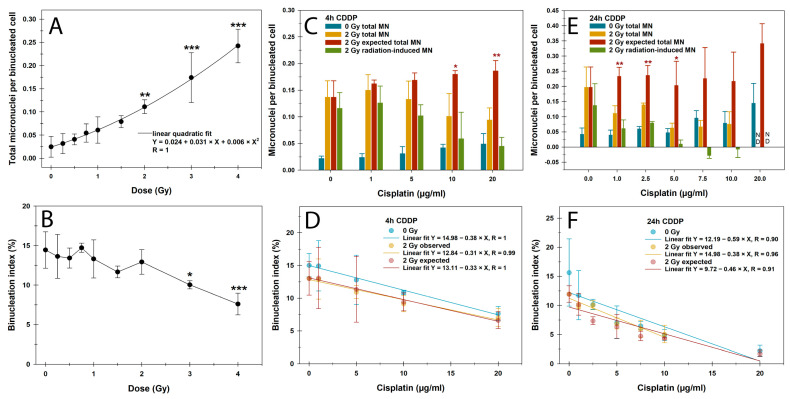
Cytokinesis-block micronucleus assay data showing (**A**,**C**,**E**) micronuclei (MN) frequencies in binucleated (BN) cells and (**B**,**D**,**F**) the binucleation index in peripheral blood lymphocytes after (**A**,**B**) X-rays or (**C**–**F**) cisplatin (CDDP) and their combination with a (**C**,**D**) 4 h or (**E**,**F**) 24 h CDDP pretreatment before 2 Gy X-ray exposure. (**C**,**E**) MN yields are presented as sham-irradiated controls without and with CDDP pretreatment (0 Gy total MN), total MN post-irradiation without and with CDDP pretreatment (2 Gy total MN), expected total MN (0.5 or 2 Gy total MN without CDDP + 0 Gy total MN with CDDP), and only radiation-induced MN without and with CDDP pretreatment (0.5 or 2 Gy total MN − 0 Gy total MN). The data are presented as mean values and standard deviations of three independent experiments with single samples. Statistics were performed by (**A**,**B**) a one-way ANOVA comparison to the mock-treated control (0 µg/mL CDDP; 0 Gy) (**C**,**E**) between observed total MN post-irradiation (2 Gy total MN) and the expected totals (0 Gy total MN + 2 Gy total MN without CDDP) using Student’s *t*-test and (**D**,**F**) and ANCOVA for the slopes of linear regressions (* *p* < 0.05; ** *p* < 0.01; *** *p* < 0.001). ND, not determined.

Our next step was to investigate the influence of CDDP on the detection of IR-induced DNA damage in the proliferation-dependent CBMN assay. Irradiation with X-ray doses of up to 4 Gy led to a linear quadratic increase in MN in BN PBLs, significantly from 2 Gy onward (Figure 3A). The binucleation index, measuring proliferation activity and cell cycle arrest from genotoxic impacts, remained stable up to 2 Gy and showed a significant reduction only from 3 Gy onward. (Figure 3B). Therefore, a 2 Gy dose was chosen for the combined treatment regimens to rule out radiation effects on cell division activity.

CDDP pretreatment for this assay was based on DNA damage results from the γH2AX/53BP1 focus assay over 4 h and 24 h, followed by incubation in a CDDP-free medium for up to 72 h. For both pretreatment regimens, MN increased with CDDP concentrations, which was more pronounced after the 24 h pretreatment, reaching significance only for the highest CDDP concentration of 20 µg/mL (*p* < 0.001) (Figure 3C,E). Similarly, CDDP pretreatment reduced the binucleation index in a largely linear concentration- and time-dependent manner, with the strongest effect in the 24 h pretreatment regimen (Figure 3D,F). The respective pretreatment with CDDP and subsequent irradiation with 2 Gy had an additive effect with a comparable negative slope, which corresponded to the expected values. Overall, no significant differences were found between the negative slopes of the linear regression lines depicted in Figure 3D,F.

**Figure 4:**
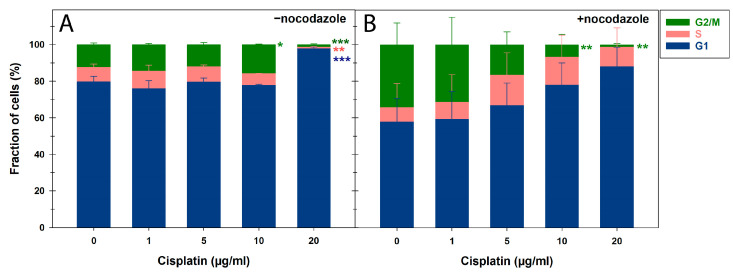
Cell cycle distribution of peripheral blood lymphocytes by flow cytometry after cisplatin treatment for 24 h in the (**A**) absence or (**B**) presence of nocodazole. The data are presented as mean values and standard deviations of three independent experiments with biological triplicates. Statistics were performed by a one-way ANOVA comparison to mock-treated cells (0 µg/mL of cisplatin) (* *p* < 0.05; ** *p* < 0.01; *** *p* < 0.001).

After the 24 h pretreatment with CDDP concentrations ≥ 10 µg/mL, the binucleation index and proliferation activity were very low and ≤5%, as confirmed by cell cycle measurements (Figure 4). Representative flow cytometric histograms of the cell cycle distribution can be found in Supplementary Figure S4. Therefore, a low concentration range of ≤10 µg/mL was applied for the 24 h CDDP pretreatment followed by 2 Gy X-rays to score MN in BN cells (Figure 3E,F). The 4 h CDDP pretreatment reduced the average total MN per BN cell after 2 Gy X-rays at CDDP concentrations of 10 µg/mL onward, though not significantly (Figure 3C). In contrast, the 24 h CDDP pretreatment significantly decreased MN frequencies in BN cells at 1 µg/mL of CDDP (*p* < 0.05) (Figure 3E). This was linked to significant underestimations of the expected MN rates following irradiation for both pretreatment regimens. Similar to the γH2AX/53BP1 focus assay, this effect caused a significant reduction in radiation-induced MN per BN cell after 24 h of CDDP pretreatment from 1 µg/mL onward (*p* < 0.05), with even negative values at higher CDDP concentrations (Figure 1E). These findings highlight a strong confounding effect of prolonged CDDP exposure on this proliferation-dependent assay for detecting IR-induced DNA damage, even after low CDDP concentrations.

## 4. Discussion

This study examined the effects of CDDP, X-rays, and their combination on PBLs from healthy donors under controlled ex vivo conditions, focusing on DNA damage, proliferation, and cell death to better understand their impact on biodosimetry and adverse hematologic treatment effects during CRT. Both treatments alone induced DSBs, micronuclei, and cell death. The combined treatment additively increased cell death and blocked mitotic progression, significantly reducing IR-related DNA damage markers. These findings highlight the adverse hematologic effects of CDDP and its confounding impact on biodosimetry during CRT.

The cytotoxic and antiproliferative actions of CDDP underlie both its therapeutic and side effects [13]. Systemic CDDP administration impacts the hematological system also for solid tumors, and its frequent combination with external beam RT can cause excessive damage to the highly radiosensitive blood system [18]. A key example is platinum-based CRT for head and neck tumor patients, where lymphopenia is a negative prognostic factor, alongside other factors, e.g., nutrition [38]. Rajkumar et al. [39] measured the concentration of CDDP in the plasma of head and neck cancer patients receiving an intravenous infusion of 40 mg/m^2^ of CDDP for 1 h or 100 mg/m^2^ of CDDP for 3 h up to 5 h or 24 h, respectively. Although free CDDP concentrations dropped significantly from peak values of ~6 µg/mL (1 h regimen) and ~11 µg/mL (3 h regimen) 1 h after CDDP discontinuation, plasma levels around 2 µg/mL remained detectable at 5 h and 24 h post-treatment, respectively. Elevated serum levels of cytostatic drugs, including CDDP, can even persist for decades after treatment and are linked to adverse long-term effects [40,41]. In this context, however, no studies exist on combined effects with civilian radiation exposures from natural background radiation or planned medical exposures. Based on Rajkumar et al. [39], we used 1–20 µg/mL of CDDP in pulse or continuous pretreatment scenarios to study its sole and combined effects with IR on PBLs under well-defined ex vivo conditions.

We first examined CDDP’s impact on the induction and repair of IR-induced DSBs, the most prompt and severe IR damage whose repair is crucial for cell survival. A 1 h CDDP pulse had no significant impact on colocalizing γH2AX/53BP1 focus yields used as DSB surrogate markers in PBLs. Only a 24 h CDDP treatment increased foci rates in a concentration-dependent manner, significantly after an additional 24 h incubation after CDDP withdrawal. Since similar initial or residual total foci counts were observed post-irradiation, both the expected levels and RIF frequencies were reduced, particularly 24 h after 2 Gy exposure from a low CDDP concentration onward. Thus, even low-dose continuous CDDP exposure can significantly impact the quantitation of IR-induced DSB surrogate markers in PBLs for biodosimetry and as predictive biomarkers in a clinical context. Whether CDDP affects the formation of IR-induced DSB repair foci in PBLs has only been studied in vivo and ex vivo by Sak et al. [33], including γH2AX as a general DSB marker and Rad51 as a marker of resection-dependent DSB repair by homologous recombination. Their studies were carried out on blood samples from 28 cancer patients with intrathoracic, pelvic, or head and neck tumors who received CDDP concurrent with RT. When patients’ blood samples were irradiated ex vivo on different days post in vivo CDDP infusion, mean γH2AX foci frequencies in PBLs dropped from about 10 to 6 per Gy between day 0 and day 4 before returning to pretreatment levels. Similarly, for the in vivo response 0.5 h after RT, with CDDP given 1–1.5 h before RT, a significant decrease in relative γH2AX foci rates in PBLs by 28% to 46% was observed between days 0 and 4 after RT. The authors also examined the sole ex vivo effect of CDDP on γH2AX foci in untreated patient samples before therapy. PBLs treated ex vivo for 1 h with up to 50 µg/mL of CDDP showed no altered foci values, aligning with our data. However, 0.5 h after 1 Gy γ-irradiation, a concentration-dependent decrease in mean foci rates was observed, starting at 1 µg/mL of CDDP. This observation is in stark contrast with our results on isolated PBLs from healthy donors, which showed no effect of a 1 h pulse pretreatment with CDDP concentrations of up to 20 µg/mL on the initial RIF rates. The reason for this inconsistency between studies remains unraveled, with the only difference being between cancer patients and healthy donors.

A reduced formation of IR-induced DSB repair foci has been generally linked to the impairment of repair mechanisms by CDDP adducts on DSBs, which prevent binding and decrease the activity of the KU70/80 heterodimer of the DNA protein kinase (DNA-PK) [10,11,42,43,44]. Abrogation of KU70/80 DSB end-binding and activity, the initiation step of NHEJ as the primary DSB repair pathway, increases lesion complexity and lethality, enhancing the combinatorial effect of CDDP and RT in tumor cell inactivation [10,44]. A major target of DNA-PK is the histone variant H2AX, which is redundantly phosphorylated at Ser139 by Ataxia Telangiectasia Mutated (ATM) and the DNA-PK catalytic subunit (DNA-PKcs) immediately after DSB induction [45]. Thus, despite potentially impaired DNA-PKcs activity in the presence of CDDP, H2AX should still be fully phosphorylated by ATM and form γH2AX foci. Additionally, using 53BP1 as another DSB marker confirmed our γH2AX observations. Despite lower RIF formation, Sak et al. [33] found no impairment in the NHEJ-mediated repair of IR-induced DSBs by CDDP in PBLs. Overall, a mechanism for the potential CDDP-related reduction in early RIF remains unraveled. Also, our data show no effect of CDDP on initial RIF levels shortly after irradiation and do not support these previous observations. However, since we withdrew CDDP before irradiation, CDDP adduct formation may have shifted toward KU70/80 binding and activity at DSBs, potentially influencing RIF formation. The effect of CDDP on the repair of IR-induced DSBs may also depend on the concentration and timing of the treatments [46].

We attribute the significant impact of the 24 h CDDP pretreatment on residual RIF levels 24 h post-irradiation to increased cell death rates. PBLs with severe pre-damage from the continuous CDDP treatment likely failed to survive additional IR-induced damage, leading to apoptosis. This is supported by higher rates of apoptotic PBLs with pan-nuclear γH2AX signals that do not allow cumulative CDDP-plus-IR-induced foci quantification. Our Annexin V/PI apoptosis measurements confirmed the mostly additive effect of CDDP pretreatment followed by IR on PBL cell death. However, we found a mild and non-significant underestimation of the expected cell death rates for the combined exposures, which were attributed to a general inter-experimental variation.

As a second endpoint of DNA damage in PBLs, we used the proliferation-dependent CBMN assay, another routine biodosimeter after radiation accidents, occupational radiation exposures, and RT in tumor patients [47,48]. Expectedly, CDDP and IR alone induced cytogenetic damage detected as MN in BN cells. Compared to IR, CDDP pretreatment for 4 h and 24 h followed by 2 Gy X-rays had a mild or a highly significant reducing impact on MN frequencies, respectively. This effect caused a significant underestimation of both the expected and radiation-induced MN rates in BN cells. In general, decreased MN yields correlated with a significant BN index decrement due to CDDP pretreatment. These observations are also clinically relevant for multimodal tumor therapies, particularly for the crucial antineoplastic activity of proliferative CD8+ T cells [49].

Dolling et al. [46] already demonstrated a significant BN index reduction in normal human fibroblasts after a 1 µg/mL CDDP pulse for 0.5 h but no enhancement of IR-induced effects with 4 Gy of X-ray irradiation 24 h later. While the CDDP treatment just before irradiation slightly increased BN cells with MN, the continuous 24 h treatment at 1 µg/mL and lower reversed this effect. These results were interpreted more as a CDDP-mediated stimulation of an adaptive response promoting the repair of subsequent IR-induced DNA damage rather than an effect on the cell cycle. Also, Azab et al. [50] examined CDDP’s cytogenetic effects, finding increased chromosomal aberrations and a significant mitotic index drop but no change in the general proliferation index at 0.4 µg/mL of CDDP in cultured PBLs. Along with our similar BN index results for higher CDDP concentrations, this was confirmed by our cell cycle measurements with or without nocodazole as a spindle inhibitor. Without nocodazole, only a high concentration of 20 µg/mL of CDDP significantly affected cell cycle distribution. With nocodazole, G2/M cell proportions decreased concentration-dependently while S-phase proportions increased, suggesting replicative stress induced by CDDP adducts. Even though oncology therapies combine CDDP and spindle inhibitors with a significant benefit for overall survival, e.g., in advanced or metastatic non-small cell lung cancer as CDDP plus vinorelbine, a combinatorial antineoplastic effect on the cell cycle has not yet been investigated [51].

Wegierek-Ciuk et al. [34] studied CDDP’s impact on IR-induced MN formation, the BN index, and apoptosis based on morphological features in PBLs from gynecologic cancer patients undergoing definitive RT or CDDP-based CRT. Similar to the experimental settings of Sak et al. [33], ex vivo γ-irradiation of blood samples from CDDP-based CRT patients with 2 Gy led to fewer MN, higher apoptosis rates, and a lower BN index in PBLs compared to definitive RT. In vivo, despite higher tumor doses, larger target volumes and genotoxic CDDP, CRT patients did not show higher MN rates during treatment than RT-only patients, again due to increased apoptosis and a lower BN index. The authors attributed these observations of CDDP’s sensitizing effect on radiation cytotoxicity, promoting an ‘overkill effect’ by eliminating severely damaged cells, aligning with our findings.

Taken together, we observed primarily additive cytotoxic and antiproliferative effects of CDDP pretreatment followed by IR on PBLs. However, potentiation and synergy could not be confirmed, despite forming the clinical basis of platinum-based CRT across various tumor types [13]. As already stressed, the mode of action of CDDP alone or with IR remains diverse, multifactorial, and still not clearly understood [13,52]. It should be noted that only relatively short CDDP and overall incubation periods were used in this study. Additionally, cell proliferation was absent or limited to a single cycle up to cytokinesis. This can greatly reduce the effects of IR and CDDP compared to conventional cytotoxicity tests, like clonogenic survival assays for tumor cells with 10–14-day incubation periods [53]. Prolonged exposure and fractionation effects also occur in vivo with CRT, enhancing treatment outcomes [4]. Here, CDDP is used neoadjuvantly or adjuvantly to shrink large tumor volumes before RT, radiosensitize them, or exert systemic effects on distant metastases via spatial cooperation [4]. However, this study did not aim to replicate these complex exposure scenarios but focused on the effects on PBLs from healthy donors and radiation biomarkers under well-defined experimental conditions ex vivo to guide future in vivo studies, which are currently underway in our laboratory.

## 5. Conclusions

Our analysis of CDDP alone or with IR on isolated PBLs under controlled ex vivo conditions showed significant effects on radiobiological and biodosimetric DNA damage and repair markers. Apoptosis induction and reduced division activity, even at low CDDP concentrations, must be considered in clinical radiation studies to prevent misinterpretation. Moreover, persistently low CDDP blood levels may have deleterious and immunosuppressive long-term effects, especially with additional radiation exposure.

## Data Availability

The datasets generated and analyzed in this study are available upon request from the corresponding author.

## Supplementary Materials

The following supporting information can be downloaded at: https://www.mdpi.com/article/10.3390/cells14100682/s1, Figure S1: Quantification of colocalized γH2AX and 53BP1 (γH2AX/53BP1) foci in peripheral blood lymphocytes (PBLs) after cisplatin (CDDP), 1 Gy X-rays, or their combination, with a 1 h CDDP pretreatment before irradiation. Figure S2: Immunofluorescence microscopy overview for γH2AX signals (green) in counterstained lymphocyte nuclei (blue) 24 h post-irradiation (2 Gy X-rays) after 24 h pretreatment with increasing CDDP concentrations. Figure S3: Representative dot plots of apoptosis measurements that show Annexin V-FITC and PI fluorescence in peripheral blood lymphocytes. Figure S4: Representative cell cycle distributions shown as histograms of PI fluorescence in peripheral blood lymphocytes.

## Abbreviations

The following abbreviations are used in this manuscript:

IR	Ionizing radiation
PBMCs	Peripheral blood mononuclear cells
PBL	Peripheral blood lymphocyte
PI	Propidium iodide
CDDP	Cis-diamminedichloroplatinum II
CT	Chemotherapy
RT	Radiotherapy
CRT	Chemoradiotherapy
LA	Locally advanced
SSB	Single-strand break
DSB	Double-strand break
NHEJ	Non-homologous end joining
ICI	Immune checkpoint inhibitors
EDTA	Ethylenediaminetetraacetic acid
PBS	Phosphate-buffered saline
FCS	Fetal calf serum
RIF	Radiation-induced foci
BN	Binucleated
MN	Micronuclei
ND	Not determined
