# Supplementary material for: Effects of Cisplatin on the Radiation Response and DNA Damage Markers in Peripheral Blood Lymphocytes Ex Vivo
Source: Cells. 2025 May 8;14(10):682. doi: 10.3390/cells14100682 (PMC12109825; doi:10.3390/cells14100682)
Supplement: Supplementary file 1 [file cells-14-00682-s001.zip › Supplements/Suppl.Fig.3.pdf]

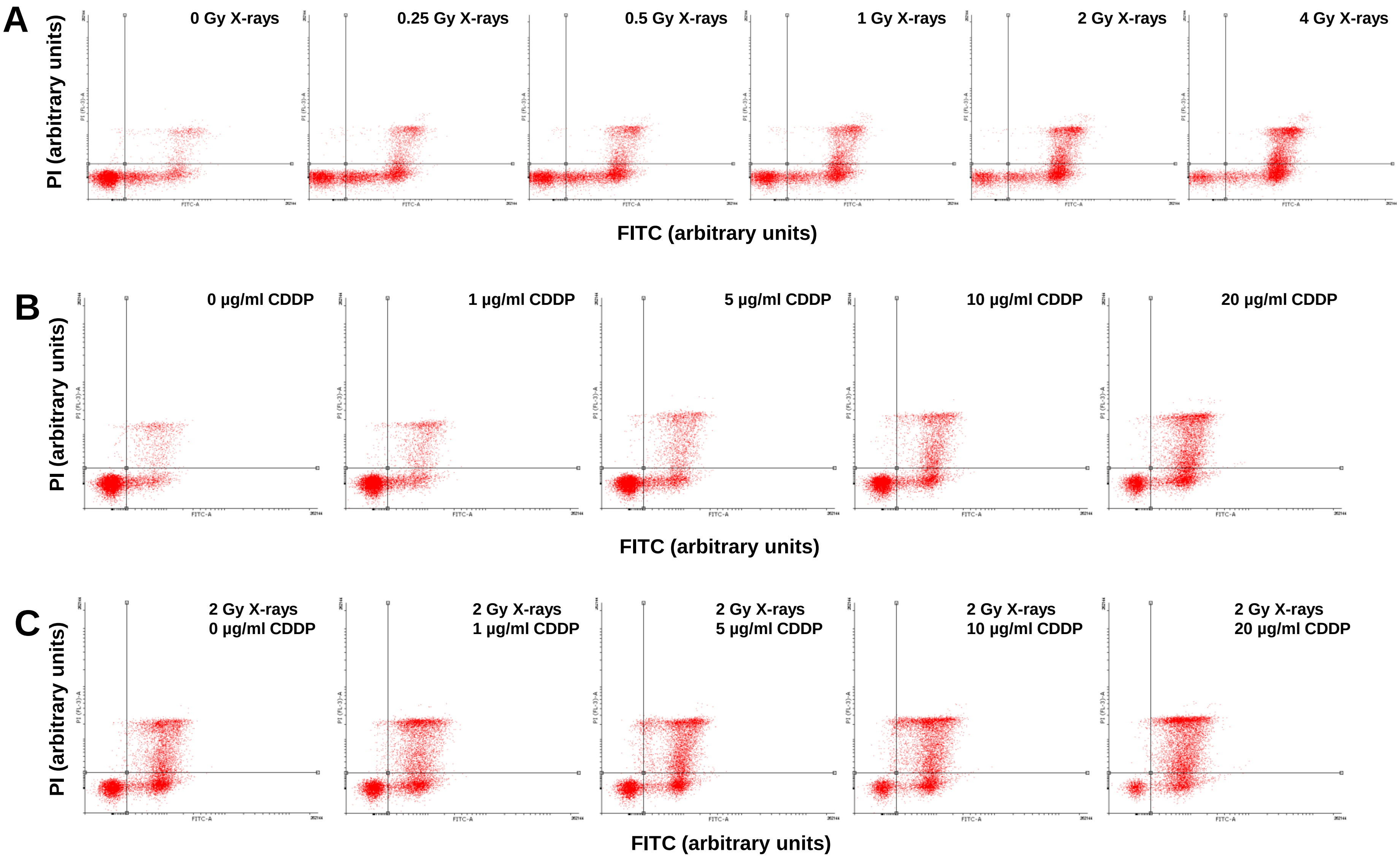

Supplementary Figure S3. Representative dot plots of apoptosis measurements show AnnexinV-FITC and PI fluorescence in peripheral blood lymphocytes 24h after (A) X-ray irradiation, (B) 24h cisplatin (CDDP) treatment, or 24h CDDP pretreatment followed by X-rays. Vital cells (AnnexinV-FITC$^-$/PI$^-$) appear in the lower left, early apoptotic cells (AnnexinV-FITC$^+$/PI$^-$) in the lower right, and late apoptotic cells (AnnexinV-FITC$^+$/PI$^+$) in the upper right quadrant.
