# Supplementary material for: Effects of Cisplatin on the Radiation Response and DNA Damage Markers in Peripheral Blood Lymphocytes Ex Vivo
Source: Cells. 2025 May 8;14(10):682. doi: 10.3390/cells14100682 (PMC12109825; doi:10.3390/cells14100682)
Supplement: Supplementary file 1 [file cells-14-00682-s001.zip › Supplements/Suppl._Fig.1.pdf]

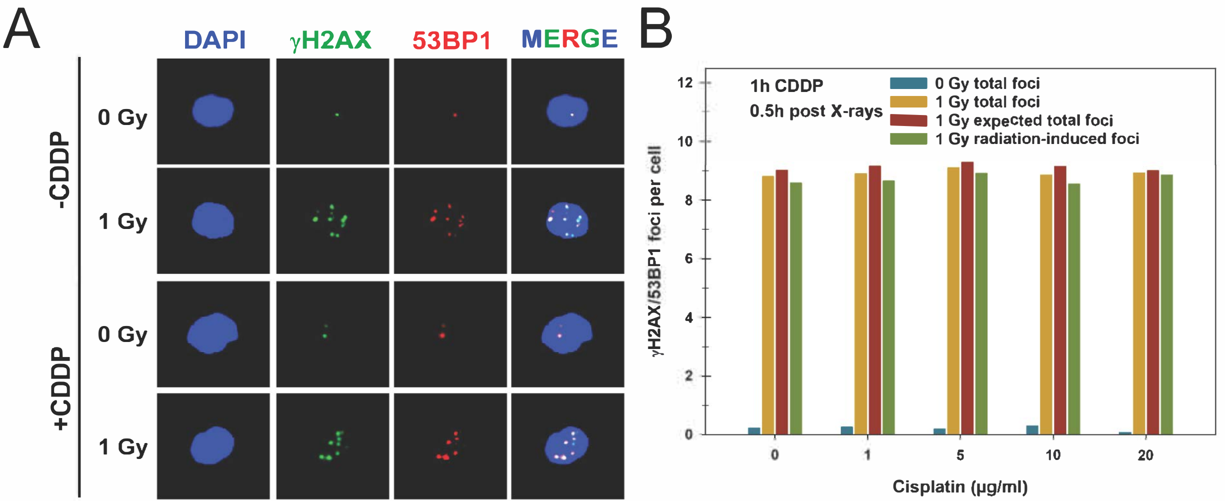

Supplementary Figure S1. Quantification of colocalized γH2AX and 53BP1 (γH2AX/53BP1) foci in peripheral blood lymphocytes (PBLs) after cisplatin (CDDP), 1 Gy X-rays, or their combination, with a 1h CDDP pretreatment before irradiation. (A) Representative fluorescence microscopic images of γH2AX/53BP1 foci in PBLs 0.5h post-irradiation, without or with 1h 10 μg/ml CDDP pretreatment. (B) Quantification of γH2AX/53BP1 foci in PBLs with a 1h pulse CDDP pretreatment 0.5h after 1 Gy X-rays. Focus yields are presented as sham-irradiated without and with CDDP pretreatment (0 Gy total foci), total foci post-irradiation without and with CDDP pretreatment (1 Gy total foci), expected total foci (1 Gy total foci without CDDP + 0 Gy total foci with CDDP), and only radiation-induced foci without and with CDDP pretreatment (1 Gy total foci − 0 Gy total foci). Data were generated in a single experiment.
