# Supplementary material for: Effects of Cisplatin on the Radiation Response and DNA Damage Markers in Peripheral Blood Lymphocytes Ex Vivo
Source: Cells. 2025 May 8;14(10):682. doi: 10.3390/cells14100682 (PMC12109825; doi:10.3390/cells14100682)
Supplement: Supplementary file 1 [file cells-14-00682-s001.zip › Supplements/Suppl._Fig.2.pdf]

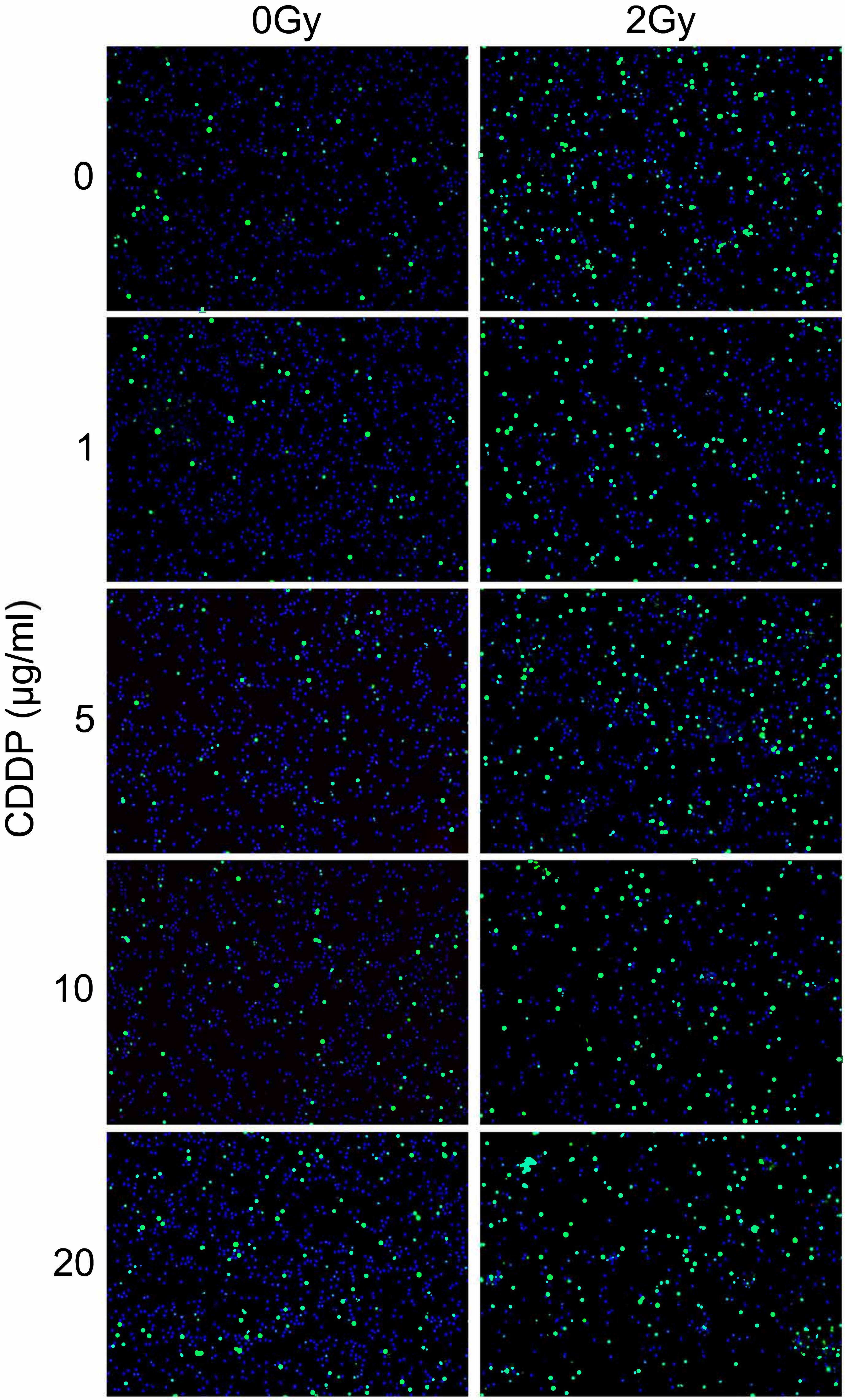

Supplementary Figure S2. Immunofluorescence microscopy overview shows γH2AX signals (green) in counterstained lymphocyte nuclei (blue) 24 hours post-irradiation (2 Gy X-rays) after 24-hour pretreatment with increasing CDDP concentrations. Bright circular green signals indicate pan-nuclear γH2AX.
