# Supplementary material for: Effects of Cisplatin on the Radiation Response and DNA Damage Markers in Peripheral Blood Lymphocytes Ex Vivo
Source: Cells. 2025 May 8;14(10):682. doi: 10.3390/cells14100682 (PMC12109825; doi:10.3390/cells14100682)
Supplement: Supplementary file 1 [file cells-14-00682-s001.zip › Supplements/Suppl._Fig.4.pdf]

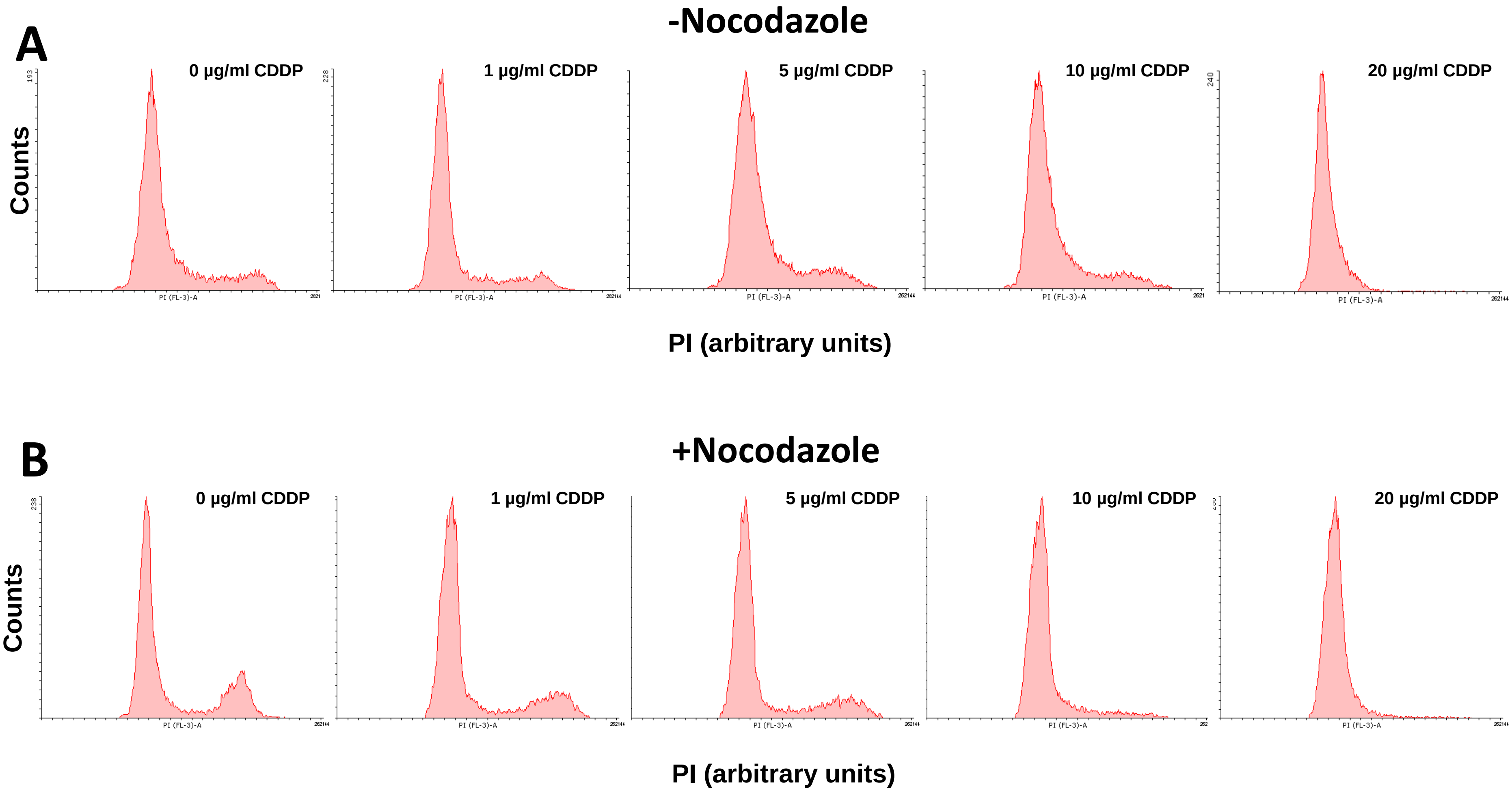

Supplementary Figure S4. Representative cell cycle distributions shown as histograms of PI fluorescence in peripheral blood lymphocytes 48h after 24h CDDP treatment (A) without or (B) with nocodazole treatment.
